# How visual perceptual grouping influences foot placement

**DOI:** 10.1098/rsos.150151

**Published:** 2015-07-08

**Authors:** John Fennell, Charlotte Goodwin, Jeremy F. Burn, Ute Leonards

**Affiliations:** 1 School of Experimental Psychology, University of Bristol, 12a Priory Road, Bristol BS8 1TU, UK; 2 Department of Mechanical Engineering, Queen's Building, University of Bristol, University Walk, Bristol BS8 1TR, UK

**Keywords:** visual perceptual grouping, locomotion, precision stepping

## Abstract

Everybody would agree that vision guides locomotion; but how does vision influence choice when there are different solutions for possible foot placement? We addressed this question by investigating the impact of perceptual grouping on foot placement in humans. Participants performed a stepping stone task in which pathways consisted of target stones in a spatially regular path of foot falls and visual distractor stones in their proximity. Target and distractor stones differed in shape and colour so that each subset of stones could be easily grouped perceptually. In half of the trials, one target stone swapped shape and colour with a distractor in its close proximity. We show that in these ‘swapped’ conditions, participants chose the perceptually groupable, instead of the spatially regular, stepping location in over 40% of trials, even if the distance between perceptually groupable steps was substantially larger than normal step width/length. This reveals that the existence of a pathway that could be traversed without spatial disruption to periodic stepping is not sufficient to guarantee participants will select it and suggests competition between different types of visual input when choosing foot placement. We propose that a bias in foot placement choice in favour of visual grouping exists as, in nature, sudden changes in visual characteristics of the ground increase the uncertainty for stability.

## 1. Introduction

Locomotion control results from large scale fusion of proprioceptive and other sensory information. In particular, vision plays a crucial role in locomotion, and how it does so has been extensively studied. Research on the visual impact on foot placement started with an influential paper by Lee *et al.* [1] on visual control of hitting the take-off board in long jump. Since then, the use of visual information to control step length has been investigated for running and walking alike [2–4]. Moreover, much is known of how vision is used to control stepping over obstacles (for recent reviews see [5,6]), avoid collisions (e.g. [7]), stepping off kerbs (e.g. [8]) and for walking on complex terrain [9–12]. Recently, it has been suggested that during visually guided walking, humans make active use of the mechanisms underlying bipedal gait by selecting the most energetically efficient footholds available, based on visual information two steps ahead [13]. We were interested here whether a largely automated sensory process in vision, perceptual grouping, would impact on foot placement. Would perceptual grouping be able to bias foot placement choice away from spatially regular placement locations expected for people walking on hard flat-level ground?

**Figure 1.**
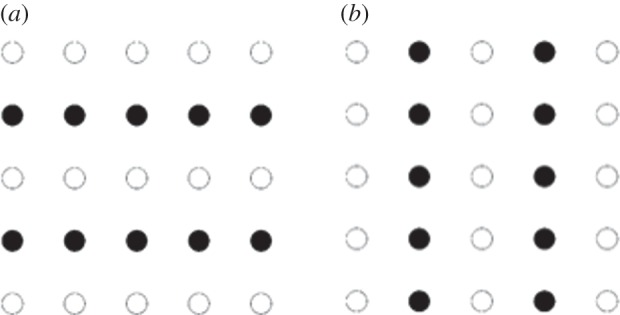
Simple examples of perceptual grouping effects, in this case grouping on the basis of similarity: the panels illustrate how changing the colour of dots alters perception from rows (*a*) to columns (*b*).

Perceptual grouping is a term used to cover a number of factors that produce well-known effects on visual perception (for review see e.g. [14]): our visual environment is perceived as consisting of organized wholes or patterns rather than individual items. Patterns are grouped on the basis of similar visual properties, such as shape or colour; proximity; continuity and symmetry (see figure 1 for an illustration). Originally investigated by the Gestalt psychologists, perceptual grouping phenomena are nowadays thought to be based on largely automatic mid-level core sensory processes involved in figure-ground segmentation and object recognition outside conscious awareness (but see [15] for the possibility of incremental grouping processes in vision). Therefore, for the purposes of the present experiment, we define locations that conform to some form of perceptually groupable properties or features as ‘visually congruent’ and distinguish them from stepping locations that are spatially regular. The latter, henceforth referred to as ‘spatially regular’ should support a stereotyped walking action at a comfortable pace for the individual, as kinematics of locomotion on a continuous hard flat-level ground are known to show low inter-stride variance [16,17]. We reasoned that spatially regular stepping locations should be sufficient to explain participants' foot placement choices. Note that both terms ‘spatially regular’ and ‘visually congruent’ refer to different types of visual information that can be selected by participants to choose where to place their feet.

**Figure 2.**
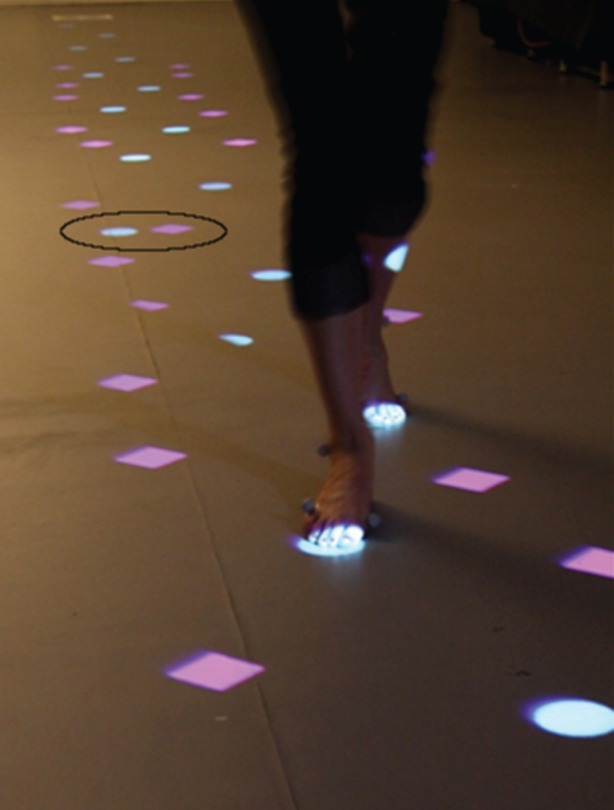
Photograph of the projected pathway for an experimental trial. For demonstration purposes, the target and distractor location of interest is here identified with a superimposed ellipse—in this example, the target location is visually incongruent, consisting of a spatially regular target with visual features otherwise found in distractors, while the distractor location close by has the visual features of the remaining target stepping stones.

In this experiment, we asked participants to follow a ‘stepping stone’ pathway that was projected onto the laboratory floor. Each projected pathway consisted of targets and distractors. Targets were considered to be spatially regular stepping locations. Distractors provided visual clutter and were projected in different shape and colour, in locations inconsistent with spatially regular stepping stones; distractors might be considered spatially irregular stepping locations. When a visual target is displayed with the same visual characteristics of the other, perceptually grouped targets, we consider the corresponding step to be visually congruent or unswapped. However, when a target is displayed with the visual characteristics of a distractor, and a distractor is displayed in the shape and colour of an otherwise visually grouped target, we consider the corresponding step to be visually incongruent or swapped. Accordingly, note that for a visually incongruent trial, a distractor could also be described as visually similar to the remaining stepping stones of interest while the target would be visually dissimilar. The distance between targets was calculated using the formula step distance=0.7×leg length in line with earlier studies [10,13] in order to provide a comfortable stepping distance for self-paced walking. Additional spatially irregular stepping locations (‘distractors’) were projected as visual clutter, or noise, on the surrounding floor (see figure 2 for an example). Participants were asked to walk at a normal walking speed along the projected pathway, taking the most direct and most comfortable route to the other end of the laboratory.

There were two experimental conditions. In one condition, the *visually congruent* or *unswapped* condition according to the definition above, all target locations were shown as the same type of visual element (e.g. a light blue/cyan circle); a second type of visual element (e.g. a pink/magenta rhombus) was consistently placed in distractor locations. In the second condition, the *visually incongruent* or *swapped* condition, all target locations were shown as the same type of visual element *except for one target location* which was presented with the visual features otherwise assigned to distractors, while the distractor location in its proximity exhibited the visual features of the normal target location. In other words, this exception step created a visual conflict between perceptually groupable but spatially irregular foot placement choice (from now on referred to as *incongruent visually similar* or *swapped visual*) and visually non-groupable but spatially regular foot placement choice (from now on referred to as *incongruent visually dissimilar* or *swapped spatially regular*). The distance between target and distractor was parametrically varied. The question we posed was to what extent, if any, would participants follow the visual information they had been primed with from the visual grouping in the reminder of the path. In other words, would participants deviate from the spatially regular stepping locations towards the perceptually groupable stepping locations? Thus, would automatic perceptual grouping impact on foot placement during walking?

Based on a substantial body of research on how vision affects locomotion during walking (for reviews see [5,9,18,19]), we predicted that the parametric variation in distance between target and distractor in the visually incongruent condition should bias participants towards preferring visually similar stepping locations (that is, distractors projected with the visual features otherwise used for targets within the path) for smaller distances, but towards spatially regular targets for larger distances. Furthermore, if participants stepped on a perceptually groupable distractor instead of a non-groupable but spatially regular target (a swapped step), in particular for larger distances between target and distractor, we expected to find a larger error in stepping accuracy (interpretable as a cost related to the irregular stepping action required for stepping on a perceptually groupable distractor), both for the step of interest as well as for the following step, analogous to increased errors observed for eye movement landing positions in the decision making literature (e.g. [20–23]).

## 2. Material and method

### 2.1 Participants

Forty-seven participants took part in the experiment (male=9). The age of the participants ranged from 18 to 66 years (*M*=31, s.d.=15.28). All the participants reported no neurological conditions that could affect mobility and walking, and all had normal or corrected-to-normal vision. None wore multi- or vari-focal glasses.

### 2.2 Materials

The experiment took place in the Bristol Vision Institute (BVI) Movement Laboratory at the University of Bristol. The laboratory is equipped with multiple projectors (Optoma EW536, resolution 1280×800, frequency 60 Hz) and a state-of-the-art motion capture system (Qualisys Motion Capture Systems, operating at 128 Hz, with a spatial resolution of approx. 1 mm). The projectors, for displaying the stimuli, and cameras for the motion capture system were mounted on metal racks surrounding the laboratory to ensure comprehensive coverage. The floor area covered by the projectors was 2 m wide×12 m long, with the motion capture system covering an area of 2 m wide×10 m (from 1 m into the projection path to 11 m of the projected path) long×2 m high. Side walls were covered with black curtain material. The mean luminance of uniform white projected onto this floor area was 4.74 cd m^−2^; and participants were dark adapted.

Each experimental trial consisted of a projected pathway comprising 16 target stepping stones together with 20 distractors. The stimuli representing the target stepping stones and distractors were shapes, circles or diamonds, coloured either light blue (cyan) (CIE *xy*:0.258/0.331; 1.96 cd m^−2^) or pink (magenta) (CIE *xy*:0.297/0.178; 0.488 cd m^−2^). Colour and shape combinations were counterbalanced for different pathways with each participant undertaking 40 trials, one trial per condition. Distractors were placed randomly from a target, at a distance of at least eight times the radius of a target, in order to minimize visual interference. However, for one of the stepping stone locations (the location of interest), the distractor was displayed (pseudo-)randomly either to the north, south, east or west of the target at a predefined distance. The predefined distance was either 25%, 30%, 35%, 40% or 45% of the participant's step length and was pseudo-randomly varied between trials. The target location of interest was placed randomly between the 9th and 13th step. A photograph from an experimental trial is shown in figure 2.

To produce walking pathways that were visually comparable between participants and for which at the same time stepping stone locations corresponded as much as possible to estimated footfall locations for walking at a self-selected comfortable walking speed, we calculated the distance between each stepping stone for each participant as 70% of their leg length [10,13] (measured from greater trochanter of the hip to floor; mean leg length 88.98±4.87 cm s.d.). The lateral distance between the centres of successive stimuli was fixed as 25% of leg length. Note, that while this distance is wider than the average step width of 13% leg length found by Donelan *et al.* [24], it means that, owing to the radius of the stimulus, the distance between the closest lateral points of successive stimuli was 15% of leg length. As a consequence, the landing area for each step has a diameter of 10% of leg length, between 15 and 25% of leg length from the nearest point of a preceding or successive stimulus. The radius of individual stimuli (target and distractor) was scaled for each participant to 5% of their leg length (in the case of diamond-shaped images, the radius was averaged).

Three 20 mm diameter spherical infrared reflectors were used per foot to enable the motion capture cameras to record foot trajectory and foot landing point. The reflectors were attached by double-sided tape to the talus, and the heads of the 1st and the 5th metatarsal. The spatial location (landing point) of the foot was measured relative to the centre of the target stepping location. The experiment used a repeated measures design with independent variables step type (congruent; incongruent), distractor location (north, south, east and west of the target location) and distance (25%, 30%, 35%, 40%, and 45% step length) as described above. Note that in subsequent analyses, we split trials for the incongruent step type into two groups, depending on whether participants stepped onto the non-groupable but spatially regular target (incongruent dissimilar/swapped spatially regular) or on the groupable distractor (incongruent similar/swapped visual).

### 2.3 Procedure

Before arrival of the participants, both visual projection and three-dimensional motion capture system were calibrated and aligned with each other. On arrival, participants provided written consent, and their leg length was measured and entered into the computer controlling the construction of the trial pathways. Then, the infrared markers were attached to participants' feet.

At the beginning of the experimental session, participants were asked to stand on a fixed starting point, and the following instructions about the experimental procedure were read out by the experimenter.

‘For this task, I would like you to walk normally across the room, using the stepping stones projected onto the floor, and get to the end of the path as directly as you can—which stepping stones you use, that is, how they look, doesn't matter.’

Note that we explicitly included the comment that the visual characteristics of the stepping stones did not matter to try to address issues of demand characteristics [25]; in other words, we tried to avoid situations in which, if participants were good at picking up the experimenter's intentions and conforming to them, they simply stuck to the perceptually grouped path because they thought that that was the main task demand.

Two practice trials and 40 experimental trials (2 [swapped/unswapped]×5 [distractor distances]×4 [distractor locations]) took place consecutively, with the participant immediately returning to the fixed starting point for the next trial unless a refreshment break was requested. On completion of the experiment, participants would sit down for the removal of the infrared reflectors, and were asked to explain what they thought the experiment had been about. After this, they were given a debriefing sheet and the opportunity to ask any questions. Participants were then thanked and escorted from the laboratory.

#### 2.3.1 Preparation of motion capture data

Data from the motion capture system were provided as three-dimensional coordinates, *x*=transverse, *y*=direction of travel and *z*=vertical, for every 1/128th of a second for every trial. Preparation of these data was required in order to identify the actual landing points and to calculate the angle and distance of the landing points from the target points. This was carried out by identifying when the forward movement of the foot is zero (or, in practice, less than a threshold value; in the present case 1 mm). The data were smoothed using a simple moving average of differences and then selecting the point of least change where the mean is less than the threshold.

## 3. Results

**Figure 3.**
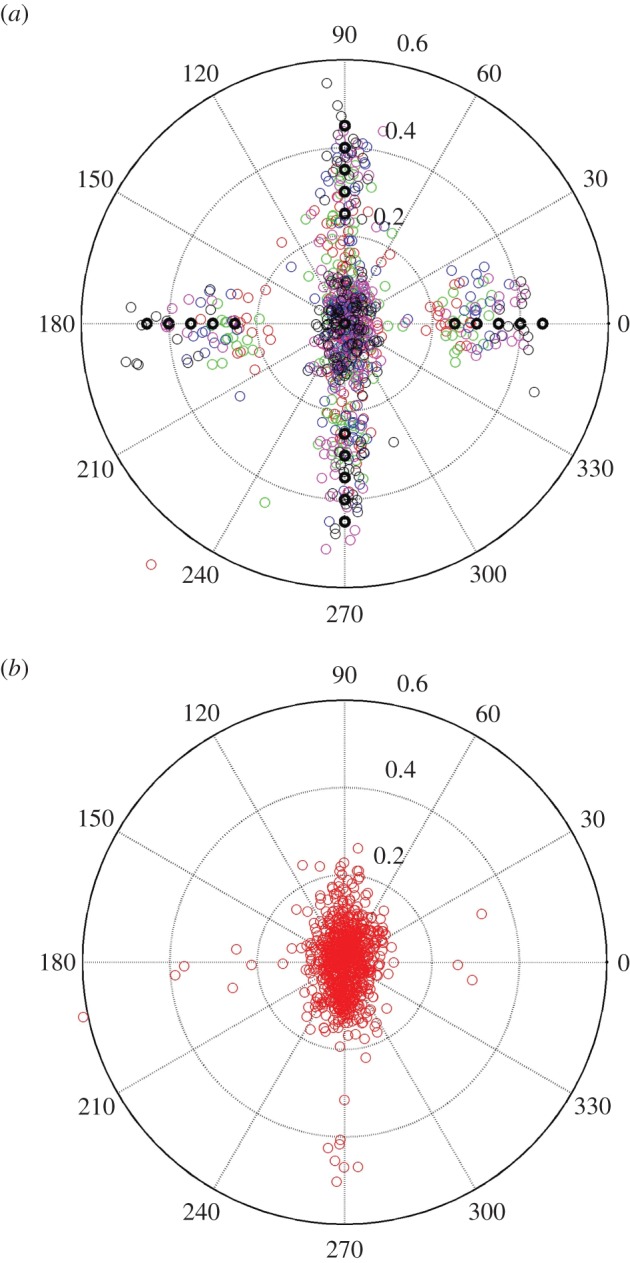
Polar plot showing the distance of the actual landing point from the target location (*a*) for visually incongruent/swapped trials and (*b*) for visually congruent/unswapped) trials. The centre of the polar plots, (0,0), represents the spatially regular target location; distractor locations (inner—0°, north—90°, outer—180° or south—270° positions) at each of the distances in step length (0.25%, 0.3%, 0.35%, 0.4% and 0.45%) are represented by the thick black circles with the actual footfalls relating to each distance shown as red, green, blue, magenta and black, respectively.

Following data preparation as described above, and accounting for practice steps and data not being correctly recorded, the maximum number of location-of-interest steps (1974=47 participants×40 trials) was reduced to 1740 (872 congruent/unswapped and 868 incongruent/swapped), corresponding to a loss of 11.8% of data for further analysis. The distance of the landing points from the centre of the chosen stepping stone was calculated for: (i) the location of interest, (ii) some control steps (steps 1–3 of the recorded path), (iii) one step before the target step (before step), and (iv) the step immediately following the location of interest (after step). A representation of the landing points (foot placements) for the target conditions are shown in the polar plot, figure 3.

From visual inspection of the top polar plot in figure 3, it is clear that a substantial proportion of footfalls to target steps for incongruent/swapped conditions were made to the visually similar distractor position and not to the spatially regular target position, in line with our hypothesis that low-level visual factors such as perceptual grouping might be able to bias foot placement decisions. However, taken together with the multiple distances between location-of-interest targets, the distribution of steps meant that we needed an objective way to quantify which steps were to targets (incongruent dissimilar/swapped spatially regular) and which were to distractors (incongruent similar/swapped visual). While we could potentially use a very simple algorithm to decide where people step, namely estimating which stepping stone, the spatially regular target or the visual distractor, was closest to the foot placement, we reasoned that such a measure might misclassify steps because of a step-to-step variability in length, width and timing of human gait that occurs even for normal obstacle-free walking on even ground [17,26]. Also, we expected inter-individual differences in foot placement on the stones that were unrelated to our question. Therefore, we quantified foot locations instead by fitting a two-dimensional Gaussian mixture model (GMM) to our data.

**Figure 4.**
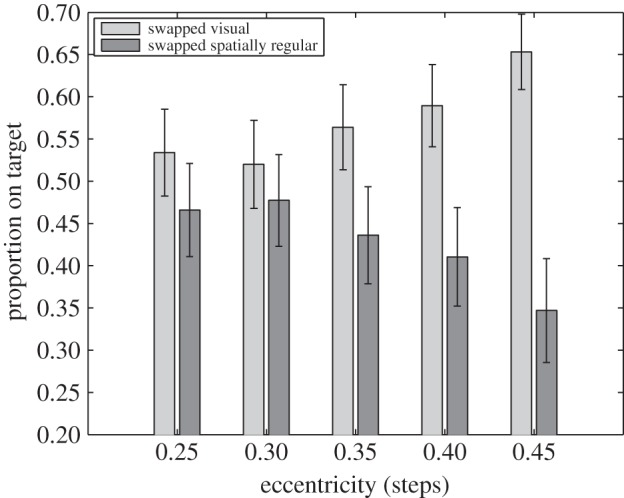
The percentage of footfalls made to visual distractors in incongruent target trials based on the fitted GMMs for each distractor distance (in step length). The light bars represent steps to spatially regular but visually dissimilar locations (swapped spatially regular), and the dark bars to visually similar locations (swapped visual). Error bars are ±1 s.e.m.

**Figure 5.**
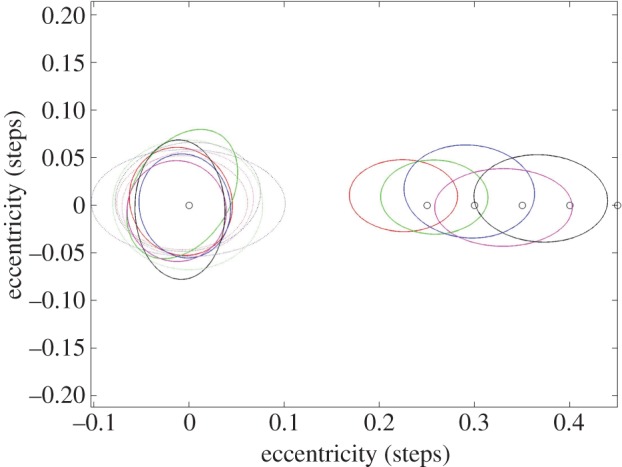
Ellipses representing 1 s.d. from the mean for the distributions of steps made to each location at each of the five stepping distances. Eccentricity for steps in the congruent (unswapped) trials is shown with dashed lines for the distributions estimated by the GMM. The eccentricity for steps in the incongruent/swapped trials are shown in red, green, blue, magenta and black, respectively, according to the displacement of the incongruent distractor stepping stone, at 0.25, 0.30, 0.35, 0.40 and 0.45 of step length, for the estimated GMM distributions. Note that footfalls have been rotated around the spatially regular target stepping stone centre so that the direction of movement of the step is always to the right of the target location—this can be seen clearly for the steps onto the visual distractor elements in the incongruent (swapped) condition, showing consistent under-stepping towards the spatially regular target.

A GMM is a probabilistic model which assumes that data points are generated from a mixture of a finite number of Gaussian distributions with unknown parameters. Fitting the best mixture of Gaussians for a given dataset (as measured by the log likelihood) results in a probability distribution of classes that can be used to predict the probability (posterior) of new data points belonging to those classes. Fitting GMMs is an example of an unsupervised learning method, and while this does not guarantee the optimal solution, models do converge quickly to a ‘local’ optimum. To improve the quality of the model, it is common practice to fit many of these models, and then choose the model that best fits the data, often on the basis of log likelihood or similar approach. In the present case, using GMM functions provided by Matlab, a mixture of two Gaussians was fitted to the data for each stepping distance. The results of this process are shown in table 1 and figures 4 and 5.

**Table 1.** Descriptive statistics for each of the distributions identified by the GMM; (*a*) overall, and (*b*) at each of the individual step eccentricities. (Incongruent/swapped trials are represented by the statistics for two distributions, labelled swapped spatially regular and swapped visual, together with before and after steps. The congruent/unswapped trials, and the steps immediately before and after the step of interest, are represented by statistics for a single distribution.)

	before			target			after								
(*a*)	*N*	m.s.e.	s.e.	*N*	m.s.e.	s.e.	*N*	m.s.e.	s.e.						
unswapped	869	0.0016	1.02×10^−4^	868	0.0038	5.05×10^−4^	864	0.0021	3.32×10^−4^						
swapped visual	368	0.0017	1.73×10^−4^	369	0.0065	4.23×10^−4^	365	0.0040	5.02×10^−4^						
swapped spatially regular	496	0.0020	3.01×10^−4^	500	0.0020	4.27×10^−4^	498	0.0021	5.25×10^−4^						

	0.25			0.3			0.35			0.4			0.45		
(*b*)	*N*	m.s.e.	s.e.	*N*	m.s.e.	s.e.	*N*	m.s.e.	s.e.	*N*	m.s.e.	s.e.	*N*	m.s.e.	s.e.
unswapped															
before	176	0.002	1.02×10^−4^	172	0.002	1.02×10^−4^	171	0.002	1.02×10^−4^	173	0.002	1.02×10^−4^	176	0.002	1.02×10^−4^
target	176	0.004	5.05×10^−4^	172	0.004	5.05×10^−4^	171	0.004	5.05×10^−4^	173	0.004	5.05×10^−4^	176	0.004	5.05×10^−4^
after	173	0.002	3.32×10^−4^	171	0.002	3.32×10^−4^	169	0.002	3.32×10^−4^	173	0.002	3.32×10^−4^	177	0.002	3.32×10^−4^
swapped visual															
before	83	0.002	2.20×10^−4^	81	0.002	2.22×10^−4^	75	0.002	3.27×10^−4^	69	0.001	3.61×10^−4^	60	0.001	7.53×10^−4^
target	82	0.003	5.30×10^−4^	81	0.003	4.97×10^−4^	75	0.005	7.02×10^−4^	71	0.005	7.32×10^−4^	60	0.005	8.85×10^−4^
after	81	0.003	7.03×10^−4^	79	0.006	1.79×10^−3^	74	0.003	8.54×10^−4^	72	0.003	5.53×10^−4^	59	0.004	1.01×10^−3^
swapped spatially regular															
before	94	0.002	2.92×10^−4^	94	0.001	2.06×10^−4^	97	0.003	1.42×10^−3^	100	0.002	2.00×10^−4^	111	0.002	2.12×10^−4^
target	94	0.002	3.26×10^−4^	94	0.003	1.48×10^−3^	97	0.001	2.27×10^−4^	102	0.001	1.40×10^−4^	113	0.003	1.36×10^−3^
after	94	0.002	8.75×10^−4^	94	0.004	1.78×10^−3^	95	0.003	1.84×10^−3^	102	0.001	1.40×10^−4^	113	0.001	1.63×10^−4^

Table 1 summarizes the results from the GMM and provides descriptive statistics for each of the conditions (congruent/unswapped, incongruent similar/swapped visual, and incongruent dissimilar/swapped spatially regular) for each of the five target distances. In figure 4, the percentage of footfalls made to visual distractors and spatially regular target stepping stones for incongruent/swapped trials, as estimated by the GMM, is shown. From this figure, it can be easily seen that, even for large distractor distances of 45% of a step, participants still followed the swapped visual distractor input instead of the incongruent but spatially regular target in more than a third of steps. However, with increasing distractor distance, the bias between visually similar versus spatially regular but visually dissimilar input seems to change in favour of the latter, in line with expectations derived that humans use visual information to choose footholds that allow a more comfortable and spatially regular action, potentially supporting recent suggestions by Matthis & Fajen [13] of visually guided optimal exploitation of the passive mechanical structure underlying the human bipedal locomotion system. This latter idea finds further support by the data presented in figure 5, where the stepping error relative to the centre of the chosen stepping location is plotted. While stepping accuracy was clearly centred around the actual centre of the stepping stone for visually congruent targets, stepping on visual distractors in the visually incongruent condition led to consistent under-stepping, perhaps reflecting some sort of cost in making this choice.

**Figure 6.**
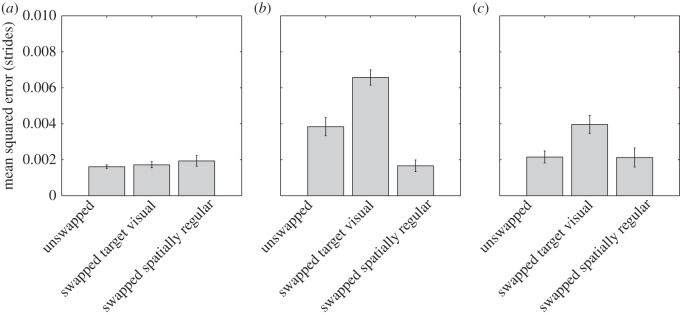
Representations of the mean squared error for (*a*) the step before the actual step of interest (before), (*b*) the actual step of interest (target), and (*c*) the step after the actual step of interest (after) for each of the two foot landing choices (swapped visual and swapped spatially regular) for incongruent trials, and for the congruent condition, averaged across all target distances. Error bars are ±1 s.e.m.

To establish the extent that stepping accuracy (i.e. the above-noted under-stepping) was affected by the choice of visual input or, in other words, to confirm whether there was competition between spatially regular stepping information and perceptual grouping information, we analysed the distance of the landing point from the centre of the stepping location participants had chosen. Analogous to saccadic eye movement accuracy to targets in the presence of distractors (e.g. [20,21]), we reasoned that, if stepping choice was affected by competition between two stepping alternatives, this should lead to a cost in stepping accuracy. As seen in figure 6*b* and statistically confirmed by a multi-way ANOVA (see below), this was unsurprisingly the case (see also figure 4). We also compared the variability of the accuracy of the step before the location-of-interest step and the step immediately after the location-of-interest steps. The former would indicate whether participants planned their steps in advance as seen for obstacle avoidance or walking on complex terrain (e.g. [10,13,27,28]); something for which no indication was found in our data (figure 6*a*). The latter would simply confirm that steps differing in length and/or width from the average step length/width impact stepping accuracy; participants' steps for elements following a step to a visually similar but spatially irregular location in a swapped trial were more variable than those following a step to a visually congruent/unswapped target or a visually dissimilar/spatially regular location in a swapped trial (figure 6*c*), which was statistically confirmed (see below).

A multi-way ANOVA with condition (congruent/unswapped, incongruent/swapped visual and incongruent/swapped spatially regular), and position (before, at and after target) as independent variables, and variance of accuracy as the dependent variable, revealed main effects of condition (*F*_2,5186_=14.95, *p*<0.001) and position (*F*_2,5186_=23.28, *p*<0.001), together with a significant interaction between condition and position (*F*_4,5186_=6.88, *p*<0.001). Tukey's test revealed a reliable difference between target steps in each condition (all *p*≤0.001). Tukey's test also revealed a reliable difference between steps to incongruent visually similar locations and the other two conditions, unswapped (*p*=0.004) and incongruent visually dissimilar/swapped spatially regular (*p*=0.008); thus confirming our hypothesis above.

## 4. Discussion

When participants were requested to step on predefined stepping stones, they often preferred less spatially regular stepping locations, if these stepping locations were visually similar with earlier stepping locations. Such stepping on visually similar, but spatially irregular stepping locations comes with a cost in stepping accuracy and leads to consistent ‘under-stepping’ in the direction of the spatially regular stepping location. Taken together, these findings suggest that, when it comes to foot placement decisions, there is direct and possibly biased competition between perceptual grouping and spatially regular visual input more consistent with the low inter-stride variance usually observed for kinematics of locomotion on a continuous hard flat-level ground (e.g. [16,17]).

Given only minimal guidance, namely to ‘walk normally across the room using the stepping stones projected onto the floor’, participants followed those elements that were consistent with spatially regular stepping and, at the same time, could be perceptually grouped. However, if there was a conflict between visual similarity and spatial regularity, participants chose, in more than one-third of steps, the visually similar, thus perceptually groupable, foothold over the spatially regular, but visually dissimilar one—even in situations in which the visually similar stepping stone was 45% of a step length away from the spatially regular foot placement, and thus let to a sequence of irregular steps with substantial increase in inter-stride variance.

Before concluding that mid-level visual processes such as perceptual grouping can indeed cause a change in stepping behaviour from that expected in the presence of a set of periodically spaced stones that could be traversed with low inter-step variance at constant speed, we first need to consider caveats. The first caveat concerns the question of whether our distractor placement was far enough away from a spatially regular foot placement to increase inter-step variance over and above that usually present and comfortable, and whether our definition of spatially regular, comfortable stepping stone locations was close enough to the stride length and width an individual would chose. Indeed, it needs to be kept in mind that our calculation of a spatially regular, comfortable foot placement for an individual was purely based on recommendations from the literature that on hard level ground, on average, participants have a steady, highly regular walking pattern that is related to a participant's leg length [10,13] and to keep visual characteristics of our floor patterns as comparable as possible across participants. In reality, the exact relationship between leg length and average step length varies across participants [24,29] and might also change with the particular walking task (free walking versus stepping on stepping stones). Moreover, each step within an individual varies slightly in length, width and timing, even for normal obstacle-free walking on even ground (e.g. [16,17,26,30]). If participants were to set their feet straight into the centre of the projected stepping stones, this would have resulted in a step width of 20% of their leg length as compared with the preferred step width of 13±3% reported in the literature [24]. Could both, a possible consistent offset from a participant's natural step length/width and their natural stepping variability have resulted in a misclassification of steps in favour of visual targets in close proximity to spatially regular targets in the swapped condition? For several reasons, we think that this is unlikely. First, both consistent offset from a participant's comfortable step length and intra-individual step variability should impact on swapped and unswapped trials alike, leading for unswapped conditions to a higher percentage of stepping on ‘distractor’ footholds, at least for distractors in close proximity (25% of a step length); this is something the results of our GMM do not suggest. Second, stepping variability inherent in the kinematics of locomotion on hard flat-level ground is approximately 2 to 3 cm in regard to step width and 1.5 to 2.5 cm in regard to step length (data taken from [30]) and thus several magnitudes below the distances induced in our samples. Third, even if an additional step variability of 25% of step length away from the average spatially regular placement as used for the closest target–distractor distance were because of noise in the locomotor system and thus had nothing to do with competition between different visual inputs, such an explanation is far less likely for the largest target–distractor distance that required a misplacement of 45% of step length. Last but not least, the steps of interest were notably less accurate when participants placed their feet onto the visually similar instead of spatially regular stepping stone in swapped trials. Moreover, we found that participants consistently understepped (i.e. foot placement erred toward the spatially regular target instead of landing in the centre of the actual stepping stone). This suggests that foot placement was affected by perceptual grouping, leading to a cost in terms of accuracy of stepping on a selected stone. This cost remained for the steps immediately following the step-of-interest, further supporting ideas of competition between different visual options for foot placements.

Even though we explicitly instructed participants to ignore stepping stone shape and colour, we primed them towards a specific colour/shape combination at the beginning of any given trial, so cannot entirely exclude the possibility that participants consciously ‘stuck’ to the perceptually grouped path, because they thought that this was what was required. We think that demand characteristics being the cause of our findings is unlikely as, when asked at the end of the session, none of our participants was able to deduce the goal of the experiment or to describe the manipulations we had tested. Also, we would have expected no impact of target–distractor distance on step choice frequency in such a case. To exclude conscious selection of perceptually grouped elements, however, future experiments could manipulate cognitive load of the task to decrease the likelihood of participants actively considering stepping stone colours when choosing their next foot placement location.

Given that we show competition between visual input favouring perceptual grouping and visual input encouraging regular stepping, then the question of why mid-level perceptual Gestalt criteria should be able to directly impact on foot placement choice in the absence of obstacles remains. Here we can only speculate. From an evolutionary perspective, an animal might want to avoid any change of visual characteristics of the ground, as outside the laboratory, such changes most probably indicate a change of material characteristics/terrain and, therefore, indicate increasing uncertainty or ambiguity with regard to stability and locomotion safety. Of course, perceptual grouping is only one of a range of mid-level visual processes that might impact on foot placement, but the general idea is clear. If the location of the next potential foot placement looks similar to the one before, then it is more predictable and presumably, safer. We must anticipate the outcome of each movement through incoming sensory information and any change in the immediate environment must evoke preparedness for further movement. In this, the ability to predict the outcome of future events is vital for effective movement and, therefore, a particularly important brain function [31,32]. Outcome prediction is at the root of every decision, weighing information we already know, what our body is capable of and what our goal is (why we move) together with incoming sensory information, following the logic: ‘I've stepped on this before and haven't fallen/slipped/tripped, so I can rely on that particular surface with known prior risk’. The complex problem of where, in space and time, foot and ground meet, must be solved and choices made accordingly, to avoid injury [33].

Walking on uneven, i.e. changing, terrain has been shown to be more energetically costly [34], and to lead to a substantial increase in muscle activity and mechanical work. Therefore, it would be interesting to investigate whether the expected energy consumption when stepping on spatially regular but visually dissimilar locations differs from stepping on perceptually groupable steps [24,29,35]? If so, higher energy consumption would be expected for stepping on spatially regular but visually dissimilar locations in swapped trials than in unswapped trials to prepare for possible stepping hazards. Future experiments might address this by recording electro-myographic and metabolic energy data [36,37] to identify changes in muscle activity related to different types of visually guided foot placement choice.

In summary, using a basic phenomenon in visual perception, namely perceptual grouping, we investigated how a perceptually grouped pathway of regularly spaced stepping stones containing, in visual terms, a single displaced step, affects kinematics of gait in an environment with visual distractors. We found that, even for the displaced step, participants followed visual input based on perceptual grouping rather than stepping on a step that would allow them to continue a spatially regular walk, in more than one-third of the trials, but not without significant error in foot placement accuracy. The observed pattern of ‘under-stepping’ points towards (biased) competition between different kinds of visual input when it comes to choosing foot placements. Our results thus show that the existence of a pathway that could be traversed without spatial disruption to periodic stepping is not sufficient to guarantee participants will select it. Future experiments will have to investigate what the consequences of such visually driven choices are for the locomotor system.

## Data accessibility

At the time this study was performed, participants consented to the data of this study being used to promote scientific knowledge and for no other purpose than research. Therefore, completely open access of the data would contravene consent and ethics approval. However, for *bona fide* researchers, the fully anonymized dataset (as tab separated value files (.tsv files) for the 3D-motion capture QTM data, and as Matlab (.mat) files for the corresponding floor projection parameters) has been deposited into the University of Bristol Research Data Repository (http://data.bris.ac.uk/data/dataset/7zl3gh7ipvm51hbg4zdzndqrv). A metadata record is openly available by the repository, with a link (email) to the Research Data team at Bristol who provide information how data can be accessed by *bona fide* researchers, and who will assess the motives of potential data reusers before granting access to the data. No authentic request for access will be refused and reusers will not be charged for any part of this process.
